# Ligation of the Brachial Artery

**Published:** 1886-08

**Authors:** N. B. Kennedy

**Affiliations:** Hillsboro, Texas


### LIGATION OF THE BRACHIAL ARTERY.

(For Daniel's Texas Medical Journal.)

*Report of a Case read before the Hill County Medical and Surgical Association, July* 14, 1886, *by N. B. Kennedy, M. D., Hillsboro, Texas.*

ON Thursday morning, July 10, 1886, I was called to see Dr. J. W. Lowery's son, the messenger informing me he had been wounded with a knife, and was about to bleed to death. In company with Dr. H. B. Vaughan, I hastened to Dr. Lowery's, who lives some eighteen miles from Hillsbro, and three miles from Aquilla Station, on the Texas Central Railroad.

On arrival, at 12 o'clock noon, Dr. Lowery sent a messenger immediately for Dr. Pratt, living one mile distant, who had seen the case the night before. Dr. Lowery gave us the following history of the case: His son was nine years of age, and had always been a bright, healthy boy. We found him a very bright, intelligent little fellow. About three weeks before this time the son was assisting the father in marking some pigs, and, in running after the pigs, he ran full tilt against the knife in his father's hand. The knife was very sharp, and had a broad blade.

The blade of the knife entered the front of the fore arm, about two inches below the bend of the elbow, completely severing the radial artery, and dividing some of the fibres of the median nerve. As an evidence there was no radial pulsation, one-half of the index finger was totally devoid of sensation and cold, while the thumb, although sensitive to the prick of a pin, yet had an abnormal burning sensation in it

The doctor, as soon as he discovered his son bleeding, immediately applied compresses soaked in tannic acid to the wound, which completely controlled all external hemorrhage. The little fellow seemed to be doing well, except that he commenced to have pain in the wound, which, at times, was very severe. There was no hemorrhage for a week or ten days, when, suddenly, the wound burst asunder; and the hemorrhage was very profuse, the blood gushing out *per saltum*. The doctor again applied his compresses, soaked in tannic acid, and again had the external hemorrhage under control. The doctor said, before applying the compresses, he noticed considerable swelling at the bend of the elbow, and that the integument in the vicinity of the wound had a bluish appearance.

On the night of June 9th, the doctor had gone to church, when a messenger was sent for him to come home post haste, as the compresses had given away, and the hemorrhage was as profuse as ever. On reaching home he found his wife had used the compresses, and had the hemorrhage under control. The doctor sent for Dr. Pratt, who came promptly. and gave the little fellow an hypodermic of a quarter of a grain of morphia to allay pain, as he was suffering with very great pain.

On removing the bandages and compresses, and washing away all externally clotted blood, we found the integument ol a bluish color, and much swelling at the bend of the elbow; and the tissues very much indurated. We decided that the swelling and hardness was due to extravasated blood, which had dissected up the tissues and clotted.

Drs. Pratt, Vaughan, Lowery and myself were decidedly and unanimously of the opinion that, instead of endeavoring to find the contracted ends of the radial, a better operation would be to ligate the brachial in the middle of the arm. So much blood had been extravasated, and the ends of the radial so much contracted, that it would have been difficult to operate successfully.

Mr. Bryant, in his admirable work on surgery, says: "This operation (referring to ligation of the radial artery) is a very difficult and uncertain one, and it is an open question whether it is a sound one. I am disposed to think the brachial had better be tied, under all circumstances, rather than have recourse to it."

Having everything in readiness that we should need for the operation, we proceeded to ligate the brachial artery of the right arm. The middle of the arm was the point of election. The course of the artery was indicated by a line drawn from the middle of the axilla to the inner side of the biceps tendon at the bend of the elbow; and the inner border ef that muscle was our guide to the incision. With the arm extended and supinated, we made a cut some two inches long. The skin and superficial fascia being very thin, we very carefully divided and exposed the deep fascia. We next proceeded, with care, to lay open the deep fascia; after which we flexed the fore arm. We found the basilic vein lying immediately below the deep fascia, on the inner side of the brachial artery, the ulnar nerve on the inner side of the vein, and the median nerve in front of the artery. We were careful not to open the sheath of the biceps muscle. After reaching the deep fascia, in which we knew the artery was imbedded (for it is well known that the trunks of all arteries—except the cutaneous—are covered in by fascia), we ceased cutting, and used the handle of the instrument; and gently tore up the fascia until the sheath of the artery was then exposed It was then carefully raised on forceps and opened—the opening being only just large enough to admit the end of an aneurism needle. The needle, armed with a catgut ligature, was passed between the vein and artery, the needle withdrawn, and the ligature tied hard and fast, and cut off short. The wound was then cleansed with a carbolized lotion; and, during every step of the operation, our hands, sponges and instruments were disinfected with a five per cent. solution of carbolic acid. The edges of the wound were brought together and sutured, and a carbolized lotion applied.

We then decided to cut down on the old wound, which we did by an incision some two and one-half inches in length, and turned out a large quantity of clotted blood. After washing out the wound, we had some hemorrhage, which was very easily controlled. We were of opinion the hemorrhage was from the inferior profunda branch of the brachial. After thoroughly cleansing the wound and noting that all hemorrhage had ceased, the edges of the wound were brought together and sutured and carbolized lotions applied.

The anæsthetic used was chloroform. The patient took it well, but was some time coming under its influence; but there was nothing denoting any untoward result. About the time we had completed the operation, and had begun to congratulate ourselves, the patient's pulse suddenly ceased at the wrist, and he ceased to breathe.

We immediately resorted to every expedient we could think of to restore animation; but with no success, when Dr. Vaughan proposed that we try the great French surgeon, Nelaton's, plan, which we did, with the happiest results.

As soon as we placed the patient's head downwards and heels up, he gasped, and soon commenced to breathe, and his pulse returned to the wrist. We remained with him until he was fully restored and had taken some food.

June 11th.—Patient had some surgical fever, the circulation being slightly accelerated—temperature 101° F. Dr. Pratt gave antipyrine, which soon reduced the temperature below normal, when quinine and stimulants were given. There had been no pain in the arm, and the patient had slept well.

June 13th.—Again saw the patient. The wound in the arm had very nearly healed by first intention, and the wound in the fore arm was granulating and discharging a laudable pus, while the general appearance of the fore arm had improved, and the dark appearance of the integument had entirely disappeared, and the member had a natural, healthy appearance. Dr. Lowery informed he had some little fever every day since the operation, but that his appetite had continued good, and that he continued to sleep well. Temperature this morning 99⅘° F., and circulation normal. The quinine had been continued from the morning of June the 11th.

From this time on the patient improved rapidly, and is now well.

